# Hydrogen sulfide provides cardioprotection against myocardial/ischemia reperfusion injury in the diabetic state through the activation of the RISK pathway

**DOI:** 10.1186/s13618-014-0020-0

**Published:** 2014-12-12

**Authors:** Jonathan P Lambert, Chad K Nicholson, Hena Amin, Sana Amin, John W Calvert

**Affiliations:** Department of Surgery, Division of Cardiothoracic Surgery, Carlyle Fraser Heart Center, Emory University School of Medicine, 380 Northyards Boulevard, Suite B, Atlanta, GA 30313 USA

**Keywords:** Diabetes, Cardioprotection, Hydrogen sulfide, RISK pathway, Myocardial Ischemia-reperfusion injury

## Abstract

**Background:**

Coronary artery disease remains the principal cause of death in patients with diabetes mellitus. Diabetic mice display exacerbated injury following myocardial ischemia-reperfusion (MI/R) and are resistant to most therapeutic interventions. We have reported that sodium sulfide (Na_2_S) therapy confers cardioprotection during MI/R in non-diabetic mice. Here we tested the hypothesis that Na_2_S therapy would limit the extent of myocardial injury following MI/R when administered at the time of reperfusion.

**Methods and results:**

Diabetic mice (db/db, 12 weeks of age) were subjected to transient myocardial ischemia for a period of 30 minutes followed by reperfusion up to 24 hours. Na_2_S (0.05 to 1 mg/kg) or saline (vehicle) was administered into the left ventricular lumen at the time of reperfusion. Na_2_S therapy significantly decreased myocardial injury in the db/db diabetic mouse, as evidenced by a reduction in infarct size and circulating troponin-I levels. The reduction in myocardial injury was also associated with a reduction in oxidative stress and a decrease in cleaved caspase-3 expression. In an effort to evaluate the signaling mechanism responsible for the observed cardioprotection, additional groups of mice were sacrificed during early reperfusion. Hearts were excised and processed for Western blot analysis. These studies revealed that Na_2_S therapy activated the Erk1/2 arm of the Reperfusion Injury Salvage Kinase (RISK) pathway.

**Conclusion:**

These findings provide important information that myocardial Erk1/2 activation by Na_2_S therapy following MI/R sets into motion events, which ultimately lead to cardioprotection in the setting of diabetes.

## Introduction

Diabetes mellitus is a disease of metabolic dysregulation characterized by abnormal glucose metabolism [1]. It is associated with a number of long-term complications associated with a decreased quality of life and reduced life expectancy including nephropathy, retinopathy, stroke and cardiovascular disease. For instance, patients with Type 2 diabetes mellitus (T2DM) have up to a 4-fold increased risk of developing coronary heart disease compared to non-diabetic patients. Moreover, patients with T2DM have a higher risk of mortality following myocardial ischemia compared with non-diabetics [2] due in part to an increased size of myocardial infarction [3]. Therefore, it is critically important to develop and implement therapeutic strategies that will attenuate myocardial infarct size in T2DM patients. However, limited basic science research has been performed in the field of acute myocardial infarction in diabetic models as the large majority of research studies investigating myocardial ischemia-reperfusion (MI/R) injury have focused on otherwise healthy animals. Moreover, of the studies that have investigated the pathophysiology of MI/R injury in diabetes the majority have predominately used Type 1 diabetic models. This is an important observation given that T2DM encompasses roughly 90% of diabetic patients [4]. As such, there is a paucity of research investigating the mechanisms of increased myocardial infarction in the setting of T2DM.

Hydrogen sulfide (H_2_S) is an endogenously produced gaseous signaling molecule that is critical for the regulation of cardiovascular homeostasis [5,6]. It is produced enzymatically in mammalian species via the action of three enzymes in the cysteine biosynthesis pathway: cystathionine-γ-lyase (CSE), cystathionine-β-synthase (CBS), and 3-mercaptopyruvate sulfutransferase (3-MST). Over the last several years, several labs including ours have investigated the therapeutic potential of H_2_S. These studies provide compelling evidence that both exogenous and endogenous H_2_S exert cytoprotective effects, especially against MI/R injury and heart failure [7-14]. These and other studies demonstrate that H_2_S utilizes a variety of effects to counter ischemic injury, including its ability to attenuate oxidative stress, inhibit apoptosis, and reduce inflammation [15]. Together, these findings suggest that therapy targeting endogenous and exogenous H_2_S offer cytoprotection against MI/R injury.

Recently, a role for H_2_S in the etiology of diabetes has been suggested [16]. More specifically, circulating levels of H_2_S are negatively related to diabetes. For instance, plasma H_2_S levels decline in response to streptozotocin [17]. Similarly, plasma H_2_S levels, as well as the aortic production of H_2_S progressively decrease as the diabetic pathology increases in non-obese diabetic mice [18]. Additionally, we found that lower levels of H_2_S are not confined to the circulation in the setting of diabetes, as evidenced by the findings that cardiac levels of H_2_S were also decreased in db/db diabetic mice. On the basis of this evidence, one can speculate that decreased H_2_S levels contribute to the pathophysiology of diabetes [19]. This postulate is further supported by the findings that restoring H_2_S levels in the setting of diabetes protects against myocardial infarction, cardiomyopathy and vascular dysfunction in models of STZ-induced diabetes [20-22]. Clinically, the negative association between diabetes and H_2_S also exists, as evidenced by the findings that lower circulating H_2_S levels are detected in plasma samples taken from patients with T2DM [16,23]. Therefore, strategies designed to restore H_2_S levels have the potential to be used as adjuvant therapy to provide beneficial effects against the cardiovascular complications associated with diabetes.

To date, the vast majority of research studies investigating the cardioprotective effects of H_2_S in models of acute MI/R injury have been conducted in non-diabetic animals. Therefore, the purpose of this study was to determine if H_2_S therapy given at the time of reperfusion could provide cardioprotection in the setting of diabetes using a well-established *in vivo* mouse model of MI/R injury.

## Materials and methods

### Animals

Male non-diabetic (C57BLKS/J) and diabetic (BKS.Cg-Dock7^M^+/+Lepr^db^/J mice; Jackson Labs, Bar Harbor, ME) were utilized at 12 weeks of age. All experimental mouse procedures were approved by the Institute for Animal Care and Use Committee at Emory University School of Medicine and conformed to the *Guide for the Care and Use of Laboratory Animals*, published by the National Institutes of Health (NIH Publication No. 86-23, Revised 1996) and with federal and state regulations.

### Materials

Sodium Sulfide (Na_2_S; Sigma Aldrich. USA; catalog# 407410) was dissolved in saline and administered using a 32-gauge needle at doses ranging from 0.05 to 1 mg/kg in a final volume of 50 μL as a single injection directly into the lumen of the left ventricle at the time of reperfusion. Saline was administered in the same manner for the respective vehicle groups. In all cases, the Na_2_S was prepared just prior to use. Groups of mice also received 1,4-diamino-2,3-dicyano-1,4-bis(2-aminophenylthio) butadiene (U0126; 0.1 mg/kg).

### Blood glucose determination

Blood obtained via a tail snip was screened using a Xtra glucose-monitoring system (Precision).

### Myocardial ischemia-reperfusion protocol and myocardial injury assessment

Surgical ligation of the left coronary artery (LCA) myocardial infarct size determination, and Troponin-I measurements were performed similar to methods described previously [9].

### Oxidative stress

The degree of lipid peroxidation was determined by evaluating the levels of malondialdehyde (MDA) in heart tissue using a commercially available thiobarbituric acid reactive substances (TBARS) assay kit according to the manufacture’s instructions (Enzo Life Sciences; catalog# ALX-850-287-KI01).

### Western blot analysis

Samples of the heart were homogenized to obtain whole cell fractions. Equal amounts of protein were loaded into lanes of polyacrylamide-SDS gels and Western blot analysis was performed as previously described [9].

### Caspase-3 activity

The activity of Caspase-3 was measured in heart homogenates using a commercially available assay kit according to the manufacture’s instructions (abcam; catalog# ab39401).

### Statistical analysis

All data in this study are expressed as mean ± standard error (SEM). Differences in data between the groups were compared using Prism 4 (GraphPad Software, Inc) with Student’s paired 2-tailed t test or one-way analysis of variance (ANOVA). For the one-way ANOVA, if a significant variance was found, the Tukey test was used as the post hoc analysis. A p value less than 0.05 was considered significant.

## Results

### Diabetes increases injury following MI/R

**Figure 1:**
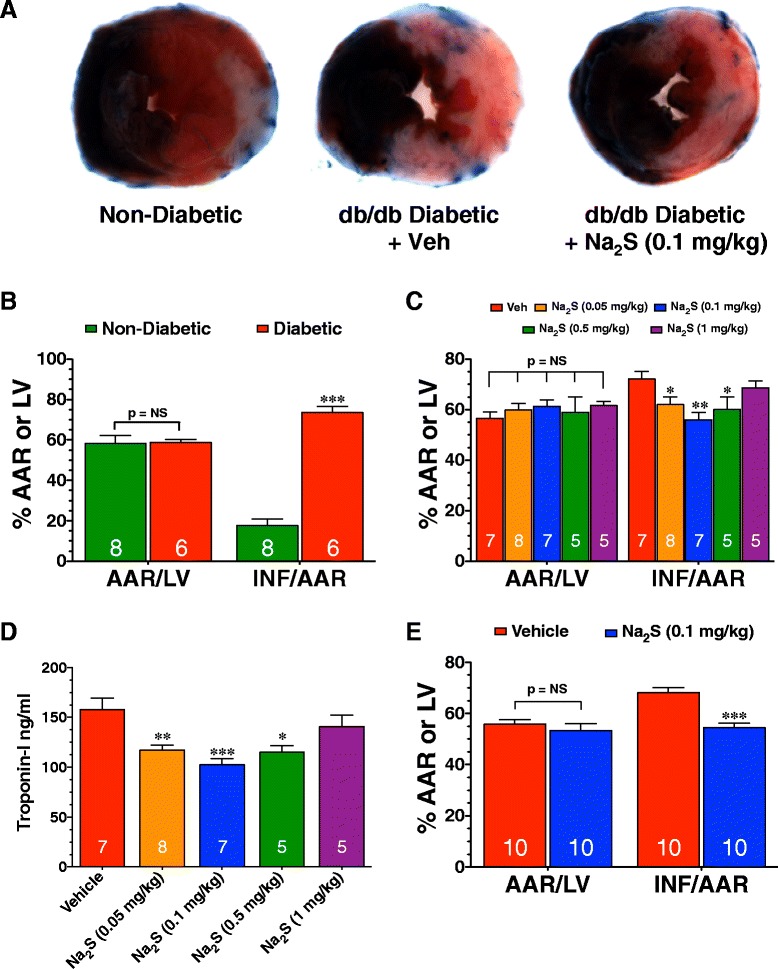
**Na**_**2**_ **S therapy reduces the extent of myocardial injury in db/db diabetic mice following MI/R. (A)** Representative midventricular photomicrographs of hearts from a non-diabetic mouse and from db/db diabetic mice treated with vehicle or Na_2_S (0.1 mg/kg) at the time of reperfusion. **(B)** Myocardial infarct size relative to the area-at-risk (INF/AAR) in non-diabetic and diabetic mice subjected to 30 minutes of ischemia followed by 4 hours of reperfusion. **(C)** Myocardial INF/AAR and **(D)** circulating troponin-I levels in db/db diabetic mice subjected to 30 minutes of ischemia followed by 4 hours of reperfusion. For these experiments, mice were treated with vehicle (Veh) or Na_2_S (0.05 to 1 mg/kg) at the time of reperfusion. **(E)** Myocardial INF/AAR in db/db mice subjected to 30 minutes of LCA ischemia and 24 hours of reperfusion. Vehicle or Na_2_S (0.1 mg/kg) was administered at the time of reperfusion. Values are mean ± SEM. Numbers inside of the bars indicate the number of animals that were investigated in each group. *p < 0.05 and **p < 0.01 vs. Vehicle; ***p < 0.001 vs. Vehicle or Non-Diabetic.

Diabetic mice exhibited the typical characteristics of a severe diabetic phenotype when compared to non-diabetic mice, including marked obesity and hyperglycemia (Table 1). In initial studies, non-diabetic and diabetic mice were subjected to 30 minutes of LCA ischemia followed by 4 hours of reperfusion, at which time the extent of myocardial infarction was evaluated. Representative mid-ventricular photomicrographs of hearts from non-diabetic and diabetic mice are shown in Figure 1A. Diabetes increased myocardial infarct size (INF) relative to the area-at-risk (AAR) by 313% (17.8 ± 3.1 for non-diabetic vs. 73.6 ± 2.9 for diabetic, p < 0.001; Figure 1B).

**Table 1:** **Body weights and blood glucose levels**

**Group**	**n**	**Body weight**	**Blood glucose**
		**(grams)**	**(mg/dL)**
Non-diabetic	8	24.3 ± 0.9	154.1 ± 3.1
Diabetic	13	47.1 ± 0.7***	544.1 ± 19.9***
Diabetic + Na_2_S (0.05 mg/kg)	8	45.5 ± 1.3***	531.6 ± 40.9***
Diabetic + Na_2_S (0.1 mg/kg)	7	46.3 ± 1.2***	581.1 ± 29.5***
Diabetic + Na_2_S (0. 5 mg/kg)	5	48.2 ± 1.6***	536.2 ± 22.8***
Diabetic + Na_2_S (1 mg/kg)	5	50.2 ± 1.8***	554.0 ± 34.1***
Diabetic + Na_2_S (0.1 mg/kg, 24 hr rep)	10	49.9 ± 1.1***	551.1 ± 36.9***

Values are means ± SEM. ***p < 0.001 vs. Non-Diabetic.

### Na_2_S dose-dependently reduces injury in diabetic mice following MI/R

To investigate if exogenous H_2_S therapy limits MI/R injury in the setting of diabetes, diabetic mice were subjected to 30 minutes of ischemia and 4 hours of reperfusion. Na_2_S (0.05 to 1 mg/kg) or vehicle was administered at the time of reperfusion via a direct injection into the LV lumen. Na_2_S dose-dependently reduced myocardial INF/AAR (Figure 1C). A dose of 0.1 mg/kg was found to be the most protective with a 22% reduction in INF/AAR (72.2 ± 2.9 for vehicle vs. 56.0 ± 3.0 for Na_2_S 0.1 mg/kg, p < 0.01). Na_2_S also reduced circulating levels of troponin-I in a dose dependent manner (Figure 1D). In separate experiments, additional groups of mice were subjected to 30 minutes of ischemia and 24 hours of reperfusion. Analogous to the earlier findings, mice receiving Na_2_S (0.1 mg/kg) displayed a 20% reduction in INF/AAR as compared with vehicle-treated mice (Figure 1E). Body weight and blood glucose levels taken prior to ischemia are shown in the Table 1.

### The reduction in myocardial injury induced by Na_2_S is associated with a reduction in apoptosis and oxidative stress

**Figure 2:**
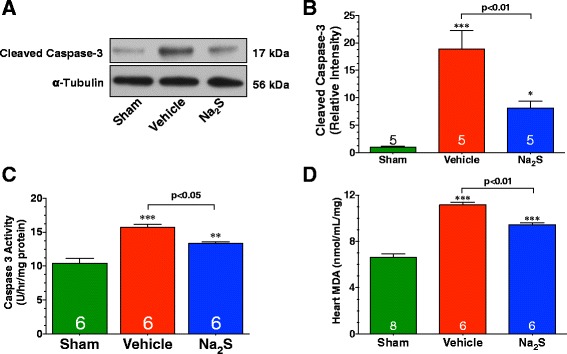
**Na**_**2**_ **S therapy reduces apoptosis and oxidative stress following MI/R.** For these experiments, the extent of apoptosis and oxidative stress was evaluated in the hearts of db/db diabetic mice subjected to 30 minutes of ischemia and 4 hours of reperfusion. Mice were treated with Na_2_S (0.1 mg/kg) at the time of reperfusion. **(A-B)** Representative immunoblots and densitometric analysis of cleaved caspase-3. **(C)** Cleaved Caspase-3 Activity. **(D)** Heart MDA levels. Values are mean ± SEM. *p < 0.05, **p < 0.01 and ***p < 0.001 vs. Sham.

Further experiments were performed to evaluate the effects of Na_2_S therapy on apoptosis and oxidative stress. For these studies, diabetic mice were subjected to 30 minutes of myocardial ischemia and 4 hours of reperfusion. Mice were either treated with Na_2_S (0.1 mg/kg) or saline (Veh) at the time of reperfusion. MI/R increased the expression of cleaved caspase-3, as well as the activity of caspase-3 in the hearts of Vehicle-treated mice (Figure 2A*-*C; p < 0.001 vs. Sham). In contrast the hearts mice treated with Na_2_S exhibited a significant reduction in cleaved caspase-3 expression and caspase-3 activity compared to Vehicle-treated mice (p < 0.05). Oxidative stress, as measured by MDA levels, was significantly increased by MI/R (Figure 2D). However, Na_2_S treated mice displayed significantly lower levels compared to Vehicle treated mice (p < 0.01).

### Na_2_S Therapy activated the Erk1/2 Arm of the RISK pathway

**Figure 3:**
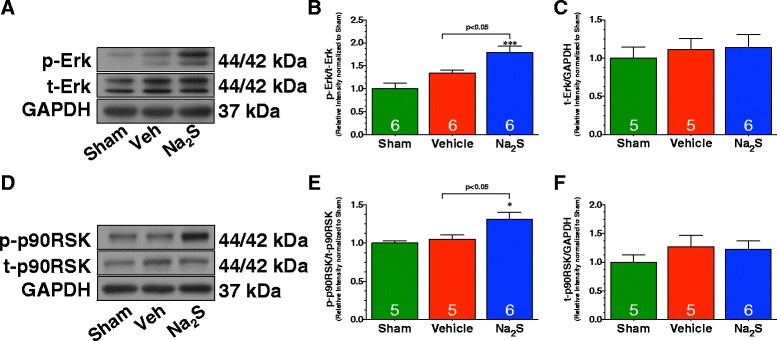
**Na**_**2**_ **S therapy activates Erk1/2 signaling following MI/R. (A-C)** Representative immunoblots and densitometric analysis of the expression of phosphorylated Erk1/2 at Threonine-202/Tyrosine-204 residues and total Erk. **(D-F)** Representative immunoblots and densitometric analysis of the expression of phosphorylated p90RSK at Threonine residue 359 and total p90RSK. Experiments were conducted with heart homogenates collected from Sham, Vehicle, and Na_2_S-treated mice following 30 minutes of ischemia and 4 hours of reperfusion. Values are mean ± SEM. *p < 0.05 and ***p < 0.001 vs. Sham.

Experiments were then conducted to elucidate potential mechanisms responsible for the cardioprotective effects of Na_2_S therapy. For these studies, we focused on components of the Reperfusion Injury Salvage Kinase (RISK) pathway, a signaling cascade involving prosurvival kinases, which confer cardioprotection when specifically activated at the onset of reperfusion following myocardial ischemia. The concept for the RISK pathway is based on the evidence that apoptosis contributes to myocyte cell death following ischemia-reperfusion injury and that activation of certain kinases exerts anti-apoptotic effects [24]. Therefore, it has been postulated that targeting these kinases at the time of reperfusion with pharmacological agents would protect the myocardium [25]. Our studies first focused on the ability of Na_2_S to activate the extracellular regulated kinase 1/2 (Erk1/2) arm of the RISK pathway. For these studies, diabetic mice were again subjected to 30 minutes of myocardial ischemia and 4 hours of reperfusion. Western blot analysis of heart homogenates collected from Sham, Vehicle, and Na_2_S treated mice revealed that MI/R did not significantly alter the phosphorylation of Erk1/2 in the Vehicle-treated mice when compared to Sham mice (Figure 3A-B). However, treatment with Na_2_S at the time of reperfusion significantly increased the phosphorylation of Erk1/2 compared to both Sham (p < 0.001) and Vehicle-treated mice (p < 0.05). Total Erk1/2 levels remain unchanged among all groups (Figure 3C).

**Figure 4:**
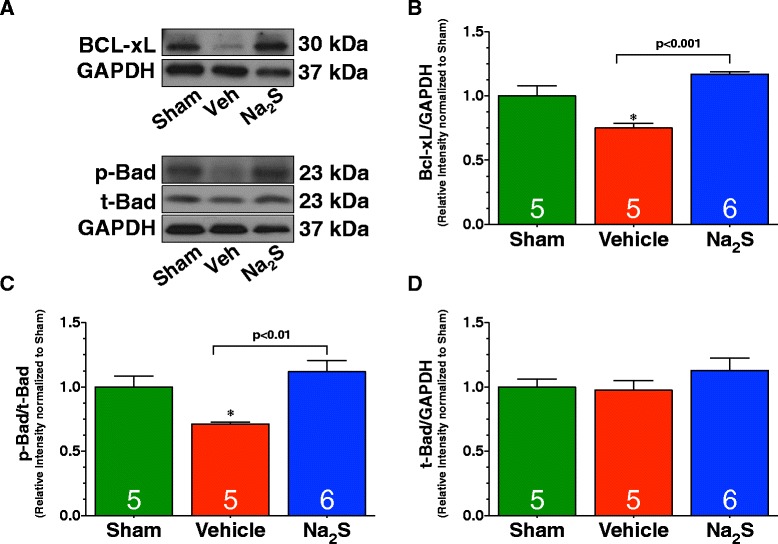
**Na**_**2**_ **S therapy increases the expression of Bcl-xL and inhibits Bad following MI/R. (A)** Representative immunoblots and densitometric analysis of the expression of **(B)** Bcl-xL, **(C)** phosphorylated Bad at Serine residue 112, and **(D)** total Bad. Experiments were conducted with heart homogenates collected from Sham, Vehicle, and Na_2_S-treated mice following 30 minutes of ischemia and 4 hours of reperfusion. Values are mean ± SEM. *p < 0.05 vs. Sham.

We then turned our attention to downstream effectors of the RISK pathway. As with Erk1/2 phosphorylation, the phosphorylation of p90RSK was only significantly elevated in the hearts of Na_2_S treated mice (Figure 3D-E; p < 0.05 vs. Sham and Vehicle). Total p90RSK remains unchanged in among all groups (Figure 3F). Next, we evaluated the expression of Bcl-xL and the phosphorylation of Bad, members of the members of the Bcl-2 family of proteins that inhibit and promote apoptosis, respectively. We chose these members because both are downstream targets of the RISK pathway. The anti-apoptotic protein Bcl-xL was found to be significantly reduced in Vehicle-treated mice compared to Sham (Figure 4A-B; p < 0.05). In contrast, Na_2_S treated mice attenuated the reduction in Bcl-xL levels (p < 0.05 vs. Vehicle). Bad can be phosphorylated at Serine residue 112 by p90RSK. Importantly, this phosphorylation site is associated with decreased apoptosis. MI/R significantly reduced the phosphorylation of Bad in Vehicle-treated mice when compared to Sham mice (Figure 4A&C; p < 0.05). However, Na_2_S treatment prevented this de-phosphorylation (p < 0.05 vs. Vehicle). Total Bad levels remain unchanged between all groups (Figure 4A&D).

### Na_2_S Therapy inhibited GSK3β

**Figure 5:**
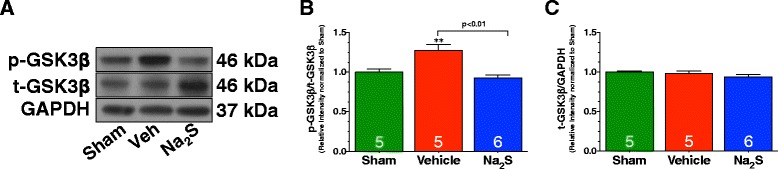
**Na**_**2**_ **S therapy inhibits GSK3β following MI/R. (A)** Representative immunoblots and densitometric analysis of the expression of **(B)** phosphorylated GSK3β at Tyrosine residue 216, and **(C)** total GSK3β. Experiments were conducted with heart homogenates collected from Sham, Vehicle, and Na_2_S-treated mice following 30 minutes of ischemia and 4 hours of reperfusion. Values are mean ± SEM. **p < 0.01 vs. Sham.

Another downstream target of both Erk1/2 and p90RSK is Glycogen synthase kinase-3Beta (GSK3β). When GSK3β is phosphorylated at Tyrosine residue 216 it is activated. Both Erk1/2 and p90RSK can inhibit GSK3β at this site via dephosphorylation. MI/R significantly increased the phosphorylation of GSK3β in Vehicle-treated mice when compared to Sham mice (Figure 5A-B; p < 0.01). In contrast, Na_2_S therapy attenuated this increase (p < 0.01 vs. Vehicle). Total GSK3β levels remain unchanged among all groups (Figure 5A&C).

### Inhibition of Erk1/2 signaling attenuated the infarct sparing effects of Na_2_S therapy

**Figure 6:**
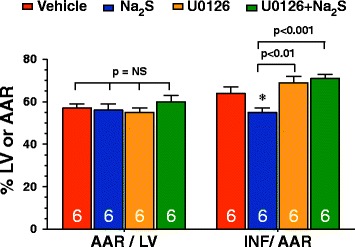
**Na**_**2**_ **S therapy attenuates MI/R injury in an Erk-dependent manner.** Myocardial INF/AAR in diabetic mice subjected to 30 minutes of ischemia followed by 4 hours of reperfusion. Mice were administered vehicle, Na_2_S (0.1 mg/kg), U0126 (0.1 mg/kg), or a combination of U0126 and Na_2_S at the time of reperfusion. Values are mean ± SEM. *p < 0.05 vs. Vehicle.

To see if the activation of Erk1/2 was necessary for Na_2_S therapy to provide its infarct sparing effects, U0126 was given alone and in combination with Na_2_S at the time of reperfusion. U0126 administration alone did not cause any further significant increase or decrease in infarct size compared to Vehicle-treated mice (Figure 6). However U0126 abolished the infarct sparing effects of Na_2_S therapy.

### Akt Signaling is not activated by ischemia nor Na_2_S therapy

**Figure 7:**
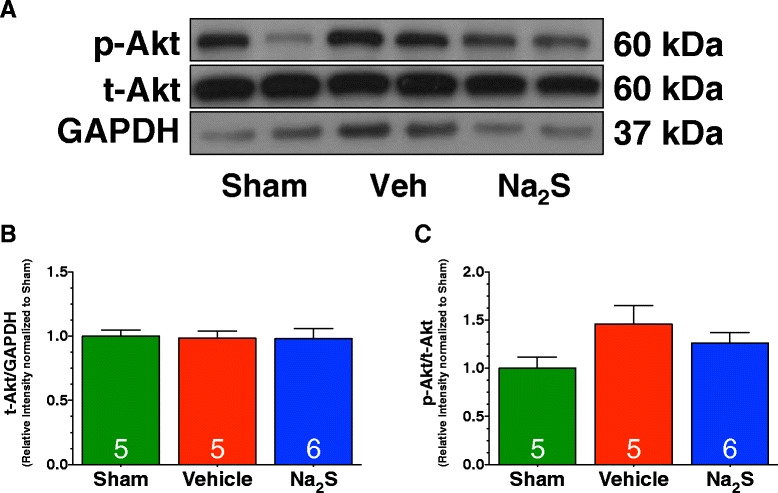
**Na**_**2**_ **S therapy did not activate Akt following MI/R. (A)** Representative immunoblots and densitometric analysis of the expression of **(B)** phosphorylated Akt at Serine residue 473, and **(C)** total Akt. Experiments were conducted with heart homogenates collected from Sham, Vehicle, and Na_2_S-treated mice following 30 minutes of ischemia and 4 hours of reperfusion. Values are mean ± SEM.

Signaling through the serine/threonine kinase Akt represents another arm of the RISK pathway. We therefore sought to determine if Na_2_S activated Akt signaling in the diabetic heart. Again, for these studies, mice were again subjected to 30 minutes of myocardial ischemia and 4 hours of reperfusion. Western blot analysis of heart homogenates collected from Sham, Vehicle, and Na_2_S treated mice revealed that MI/R did not significantly alter the phosphorylation of Akt in the Vehicle-treated mice when compared to Sham mice (Figure 7). Our analysis revealed that Na_2_S therapy also did not alter the phosphorylation of Akt. Total Akt levels remain unchanged among all groups.

## Discussion

The main findings of this study are the following: 1) Na_2_S therapy administered at the time of reperfusion reduces MI/R injury in the setting of T2DM; 2) Na_2_S therapy activates the Erk1/2 arm of the RISK pathway; 3) Erk1/2 signaling increases anti-apoptotic proteins and inhibits the activation of GSK3β 4) Na_2_S therapy provides it’s infarct sparing effects in an Erk1/2-dependent manner.

The previous studies investigating the cardioprotective effects of H_2_S have provided important mechanistic insights into its cytoprotective actions [15]. However, these studies have offered very little insights into the ability of H_2_S to protect in the setting of a diseased state, such as diabetes. As such, the results of the current study are the first to demonstrate that the administration of Na_2_S therapy at the time of reperfusion provides cardioprotection in the setting of T2DM. In agreement with a previous study with non-diabetic mice [7], we found that Na_2_S dose-dependently reduced myocardial injury, as evidenced by a reduction in infarct size and circulating troponin-I levels. However, despite the observed cardioprotective effects of an acute administration of Na_2_S in the current study, we found that the magnitude of infarct size reduction was significantly less than that observed in non-diabetic mice. This suggests that the underlying pathology present in the setting of T2DM may impair critical cardioprotective signaling and may minimize the therapeutic benefit of pharmacological agents. However, these results are of clinical relevance since they demonstrate that H_2_S treatment can potentially be initiated at the time of coronary artery reperfusion to diabetic patients experiencing myocardial ischemia in an effort to reduce myocardial infarction.

One of the major therapeutic targets for protection against MI/R injury is the activation of the RISK pathway. As noted above, the RISK pathway is a term given to a signaling cascade involving prosurvival kinases, which confer cardioprotection when specifically activated at the onset of reperfusion following myocardial ischemia. The original members reported to be a part of the RISK pathway were the phosphatidylinositol-3 kinase (PI3K), Akt, and extracellular regulated kinase 1/2 (Erk1/2). Additional studies have expanded this list to include other kinases such as protein kinase C (PKC; primarily the PKC-ε isoform), protein kinase G (PKG), and glycogen synthase kinase 3β (GSK-3β) [24,25]. It has been suggested that targeting the RISK pathway with pharmacological agents may be a viable treatment option for MI/R injury. For instance, it has now been demonstrated in preclinical models that insulin, urocortin, atorvastatin, bradykinin, opioid receptor agonists, atrial natriuretic peptide (ANP), and Glucagon-Like Peptide-1, reduce myocardial infarct size when administered at the time of myocardial reperfusion through the activation of the RISK pathway [24]. Importantly, it has also been demonstrated that ANP reduced infarct size, improved left ventricular function, and lowered combined end-point of death or cardiac failure when administered to patients undergoing primary percutaneous coronary intervention [26].

Mitochondria are essential for cell survival, both because of their role as metabolic energy producers and as regulators of programmed cell death [27]. Under normal conditions, the mitochondrial network of the myocyte must have properties of both constancy and flexibility, first providing a steady supply of ATP to fuel contraction, and second, to adapt the rate of energy production to meet the changing metabolic demand as workload varies [28]. The mitochondrial permeability transition pore (mPTP) occupies a fundamental role in determining cellular survival in the setting of myocardial ischemia-reperfusion injury because MPTP opening also causes mitochondrial membrane potential (Δ*Ψ*_m_) depolarization [28]. Early reperfusion following ischemia represents a period when Δ*Ψ*_m_ is most likely to become unstable due to the production of high levels of ROS and ensuing oxidative stress. As a result, the loss of Δ*Ψ*_m_ during this time causes a rapid impairment of mitochondrial function, which ultimately leads to apoptotic cell death through the release of pro-apoptotic proteins or can initiate necrotic cell death. Thus, maintaining Δ*Ψ*_m_ is of paramount importance during the period of early reperfusion, as it is a major determinant of cell fate following ischemia [28]. Given, that mitochondria lie at the core of existence of cellular life, it is of no surprise that they are the most common effector for numerous cardioprotective-signaling cascades. Importantly, a common target of the signaling activated by the RISK pathway is the mitochondria. Specifically, activation of the RISK pathway has been shown to inhibit the opening of the mPTP [29], thereby preventing apoptotic death caused by mitochondrial membrane permeabilitization [30].

While the downstream effectors of the RISK pathway have not been fully elucidated, the Erk1/2 signaling arm has been shown to signal through signal transducer and activator of transcription 3 (STAT-3), p90RSK, Bcl-2, Bcl-xL, and HSPs [15,25,31]. Erk1/2 dependent p90Rsk activation is essential to providing protection against reperfusion therapy because active p90RSK phosphorylates and inhibits the pro-apoptotic protein BAD [32]. This is important, because when BAD is active it binds to BCL-xL and disrupts the BCL-xL/Bax complex causing the accumulation of Bax in the mitochondrial membrane, which results in apoptosis. Additionally, Erk1/2 dependent p90Rsk activation also suppresses the opening of the mPTP by inhibiting GSK3β [33]. It is already known that the diabetic state impairs the activation of the RISK pathway in the setting of MI/R. In a type 1 diabetic rat model (streptozotocin-induced), erythropoietin (EPO)-induced cardioprotection through RISK signaling was lost [33]. Interestingly in the same study, EPO-induced cardioprotection through RISK signaling was still maintained in high fat diet (HFD)-induced insulin resistant mice suggesting that it remains unclear how different forms of diabetes and insulin resistance are affecting the activation of the RISK pathway in response to MI/R. For this reason alone it is very important to investigate how the T2DM state would affect RISK signaling and whether Na_2_S therapy would influence the RISK pathway in this model. In our previous paper we showed that 7 days of Na_2_S treatment in a preconditioned state can activate Erk1/2 [20], and because Erk1/2 is an important part of providing protection in the early reperfusion state we wanted to see if Na_2_S could activate Erk1/2 when it was administered at the time of reperfusion. The findings of the current study agree with many others that the diabetic state impairs the RISK pathway. Specifically, MI/R injury failed to activate Erk1/2 signaling in untreated mice. This was further associated with the activation of Bad and GSK3β. Based on the evidence that MI/R activates Erk1/2 signaling in non-diabetic animals [34], it can be suggested that impaired signaling in the Erk1/2 arm of the RISK pathway contributes in part to the enhanced injury observed in the db/db diabetic heart. Importantly, our findings indicate that Na_2_S therapy is able to provide protection against MI/R injury through its ability to activate Erk1/2 signaling during early reperfusion. Moreover, our findings suggest that the activation of Erk1/2 signaling and subsequent activation of p90RSK, inhibition of Bad, and inhibition of GSK3β are responsible for the anti-apoptotic and infarct lowering effects of Na_2_S therapy.

Another important finding of the study relates to the other arm of the RISK pathway: Akt pathway. Specifically, our data suggests that Akt is not activated in the db/db heart by ischemia-reperfusion injury nor Na_2_S therapy. The former is supported by previous data indicating that Akt is not activated by myocardial ischemia in the db/db heart and likely reflects an impairment in pro-survival signaling induced by diabetes. The latter is in contrast to previous findings by our group demonstrating that H_2_S therapy activates Akt in the setting of heart failure [12]. It is important to note that our previous study used non-diabetic mice and evaluated the activation of Akt in response to pressure-overload heart failure. Additionally, we evaluated the activation of Akt 6 weeks after the induction of heart failure. Therefore, it is possible that Na_2_S therapy could have an effect on Akt activation in the db/db heart at a different time point than the one we evaluated (i.e. 30 minutes of reperfusion or 24 hours of reperfusion). As such, future studies are necessary to determine the role Akt plays in mediating the cardioprotective effects of H_2_S therapy in the setting of type-2 diabetes.

Although the current study demonstrates that a single administration of Na_2_S therapy is able to reduce infarction in the setting of MI/R injury, there are some limitations that need to be noted. Because a mouse model was used, these data may not accurately predict human disease. Therefore, future studies need to be conducted in large animal models that are more clinically relevant. Another limitation is that we did not evaluate why T2DM impairs the RISK pathway during the early reperfusion period following myocardial ischemia. Future studies are definitely warranted to delve further into the mechanism(s) responsible for this impairment. Additionally, further studies are needed to evaluate how Na_2_S therapy affects the permeability of the mPTP in the diabetic state following MI/R injury.

In summary, our findings demonstrate for the first time that exogenous administration of Na_2_S attenuates MI/R injury in diabetic animals when administered at the time of reperfusion. This is important because it confirms the potential therapeutic effects of H_2_S in treating a heart attack in the setting of diabetes. It also highlights the complexity of therapeutic intervention for the diabetic heart following ischemia, as even at its most protective dose, the robust cardioprotective effects of Na_2_S that have previously been reported in the non-diabetic state were diminished in the diabetic heart.
